# Biological and Chemical Insights of Beech (*Fagus sylvatica* L.) Bark: A Source of Bioactive Compounds with Functional Properties

**DOI:** 10.3390/antiox8090417

**Published:** 2019-09-19

**Authors:** Corneliu Tanase, Andrei Mocan, Sanda Coșarcă, Alexandru Gavan, Alexandru Nicolescu, Ana-Maria Gheldiu, Dan C. Vodnar, Daniela-Lucia Muntean, Ovidiu Crișan

**Affiliations:** 1 Department of Pharmaceutical Botany, University of Medicine, Pharmacy, Sciences and Technology, 540139 Târgu Mureș, Romania; sandacosarca31@gmail.com; 2 Department of Pharmaceutical Botany, “Iuliu Hațieganu” University of Medicine and Pharmacy, Cluj-Napoca 400337, Romania; alexandru_s_nicolescu@yahoo.com (A.N.); Gheldiu.Ana@umfcluj.ro (A.-M.G.); 3 Department of Medical Devices, “Iuliu Hațieganu” University of Medicine and Pharmacy, Cluj-Napoca 400439, Romania; gavan.alexandru@umfcluj.ro; 4 Department of Food Science, Faculty of Food Science and Technology, University of Agricultural Sciences and Veterinary Medicine, 400372 Cluj-Napoca, Romania; dan.vodnar@usamvcluj.ro; 5 Department of Analytical chemistry and Drug analysis, University of Medicine, Pharmacy, Sciences and Technology “, 540139 Târgu Mureș, Romania; daniela.muntean@umfst.ro; 6 Department of Organic Chemistry, “Iuliu Hațieganu” University of Medicine and Pharmacy, 400012 Cluj-Napoca, Romania; ovicrisan@yahoo.com

**Keywords:** antioxidant, antibacterial, antifungal, antimutagen, *anti*-α-glucosidase, *anti*-tyrosinase, beech bark, microwave assisted extraction, optimization

## Abstract

The present study aimed, on the one hand, to improve the yield of microwave assisted extraction (MAE) of polyphenols from beech bark by using a design of experiments (DoE) approach. On the other hand, beech bark extracts (BBE) were characterized in terms of their phytochemical profile and evaluated for biological potential (antioxidant, antibacterial, antifungal, antimutagen, *anti*-α-glucosidase, and *anti*-tyrosinase). The extraction time varies with the amount of extracted total phenolic content (TPC). The microwave power favors TPC extraction but in different proportions. The optimum conditions which gave the highest TPC (76.57 mg GAE/g dry plant material) were reached when the microwave power was 300 W, extraction time was 4 min, and the solvent was an ethanol–water (50:50) mixture. The practical value of TPC after a controlled experiment was 76.49 mg GAE/g plant material. The identified compounds were vanillic acid, gallic acid, epicatechin, catechin, protocatechuic acid, chlorogenic acid, ferulic acid, and isoquercitrin. The antioxidant potential of BBEs was demonstrated by *in vitro* experiments. The BBEs were active against *Staphylococcus aureus*, *Pseudomonas aeruginosa*, *Salmonella typhimurium*, *Escherichia coli*, and *Candida* species. All extracts were antimutagenic and expressed an inhibition on α-glucosidase and tyrosinase activity. Regarding antimutagen activity, the assayed extracts may be considered to have low or no antimutagen effects.

## 1. Introduction

Bark plays an important role in protecting woody vascular plants, especially through its content in bioactive compounds with an antimicrobial effect [1]. The bark of woody plants is considered to be a by-product of the forestry and wood industry. This can be an important source of bioactive compounds with a high recovery potential. Numerous studies show the importance and value of bark. Moreover, bark extracts can have biological effects, such as antioxidant, antibacterial, anti-inflammatory, anti-tumor, etc., [2,3,4,5,6]. It has been determined that the bio-activity of bark natural extracts is mainly due to their content in phenolic compounds [7,8].

The beech (*Fagus sylvatica* L.) is one of the most widespread woody vascular plants in Europe and particularly in Romania, with a high economic value [9]. Beech wood is mostly used for fire wood or in the wood processing industry. After processing beech wood, a significant amount of bark is obtained.

Some literature data highlights that beech bark can be a rich source of bioactive compounds [10,11]. Furthermore, in a previous study some of the phenolic compounds obtained from beech bark by hot water extraction were identified (vanillic acid, catechin, taxifolin and syringin) [11,12]. Hofmann et al. identified 37 compounds in beech bark extracts, including catechin, epicatechin, quercetin, taxifolin, procyanidins, syringic acid, and coumaric acid [12]. Regarding the biological activity of beech bark extracts, literature information is quite limited. In previous works, the antibacterial and antitumor activity of beech bark aqueous extracts, obtained by classical extraction, was evaluated [11,13]. Beech bark extracts induced a decrease in A375 melanoma cell viability [13] and antimicrobial activity against *Staphylococcus aureus* including methicillin-resistant strains [11]. In another study, Hofmann et al. [10] found that the most efficient antioxidants in beech bark were the (+)-catechin, procyanidin B dimer 2, (−)-epicatechin, and coniferin isomer 2. Consequently, the research directions are oriented towards the identification and isolation of the bioactive compounds and the description of their mechanisms of action at the level of living organisms, finally with possibility of exploitation and functionalization on an industrial scale.

The main objectives of the current study were: (1) to optimize the extraction yield of phenolic compounds from beech bark (BB) based on an original experimental design; (2) the characterization of the phytochemical profile of optimized beech bark extracts (BBE); (3) the evaluation of the biological potential (antioxidant, antibacterial, antifungal, antimutagen, and enzyme inhibitory activity) of the optimized BBE.

## 2. Materials and Methods

### 2.1. Chemicals

The reagents used in this study were acetone, ethanol, methanol, hydrochloric acid (37%), and Folin-Ciocâlteu reagent, all of which were purchased from Merck (Darmstadt, Germany).

The standards used for both spectrophotometric and LC-MS/MS analysis were: quercetin (≥95%), hyperoside (quercetin 3-D-galactoside, ≥97%), isoquercitrin (quercetin 3-D-glucoside, ≥98%), quercitrin (quercetin 3-rhamonoside, ≥78%), (+)-catechin (≥96%), (−)-epicatechin (≥90%), vanillic acid (≥97%), syringic acid (≥95%), protocatechuic acid (3,4-dihydroxybenzoic acid, ≥97%), campesterol (~65%), ergosterol (≥95%), and stigmasterol (~95%) purchased from Sigma-Aldrich, gallic acid (≥98%) purchased from Merck (Darmstadt, Germany), and beta-sitosterol (≥80%) purchased from Carl Roth (Karlsruhe, Germany).

### 2.2. Plant Sample

The beech (*Fagus sylvatica* L.) bark samples were collected from Gurghiu Mountains, Toplița region, Mureș County, Romania, during November and December 2017. The age of the test trees was about 15–20 years. Only the bark was collected from the stems of the beech trees and (2.0 kg each) splintered manually. The species was identified and authenticated by Dr. Corneliu Tanase from the Department of Pharmaceutical Botany, the first author of the manuscript. The beech bark was air-dried at room temperature (10.5% humidity) and milled in a GRINDOMIX GM 2000 mill to a mean particle size diameter of 0.5 mm. The biomass was directly used without any pre-treatments.

### 2.3. Extraction

The extraction process was carried out based on a DoE (Design of Experiments) developed using Modde software, version 11.0 (Sartorius Stedim Data Analytics AB, Umeå, Sweden). The D-optimal design type, which is based on the selection of the experiments so that they are spread over the largest area of the variability matrix, allowed the study of two quantitative factors which varied on different levels, namely the microwave power, with 4 levels of variation (300, 450, 600, 800 W) and the extraction time, varied over 3 levels (2, 3, 4 min). The amount of extracted total phenolic content (TPC) using different solvent types (water, ethanol–water 50:50 and 80:20) was introduced in the DoE as 3 separate responses, which allowed a good comparison of the solvents’ TPC extraction capacities (Table 1).

Bark was weighed (2 g) and mixed with 20 mL of solvent (water, 50:50 ethanol–water, 80:20 ethanol–water) in Falcon tubes. The Microwave extraction (MAE) was performed using a domestic microwave oven (Samsung MS23K3513). 7. After extraction, solvent was added to give a final volume of 20 mL. Each tube was centrifuged (Hettich, Micro 22R, Andreas Hettich GmbH & Co., Tuttlingen, Germany) 15 min at 3000 rpm, maintaining the extraction temperature. The supernatant was carefully separated, and the solvent was removed under vacuum at 40 °C using a rotary evaporator (Hei-VAP, Heidolph Instruments GmbH & Co., Schwabach, Germany).

### 2.4. Quantitative Determinations of Total Phenolic Content

The total phenolic content (TPC) of the BBE extracts was determined by Folin-Ciocâlteu spectrophotometric method according to a method described previously [14]. Gallic acid was used as a reference standard, and the content of phenolics was expressed as gallic acid equivalents (GAE) per gram of dry plant material (mg GAE/g dry plant material).

### 2.5. Phytochemical Analysis by LC-MS/MS

For the description of bark extracts’ phytochemical composition a validated analytical method of liquid chromatography tandem mass spectrometry was employed (LC-MS/MS). The analysis was performed using an Agilent 1100 HPLC Series system (Agilent, Santa Clara, CA, USA) which was equipped with binary gradient pump, degasser, auto sampler, column thermostat, and UV detector. The LC system was coupled with an Agilent Ion Trap 1100 SL mass spectrometer (LC/MSD Ion Trap VL).

The LC-MS/MS analytical method was previously developed and validated [15,16] and was used for the identification of 18 polyphenols in BBE samples: caftaric acid, gentisic acid, caffeic acid, chlorogenic acid, *p*-coumaric acid, ferulic acid, sinapic acid, hyperoside, isoquercitrin, rutin, myricetol, fisetin, quercitrin, quercetin, patuletin, luteolin, kaempferol, and apigenin. For the chromatographic separation of polyphenols, a reverse-phase analytical column was used (Zorbax SB-C18, 100 mm × 3.0 mm i.d., 3.5 µm). The mobile phase was a mixture of methanol: acetic acid 0.1% (v/v) and a binary gradient was used. The elution started with a linear gradient, beginning with 5% methanol and ending at 42% methanol for 35 minutes; then isocratic elution followed for 3 min with 42% methanol. For the chromatographic data processing, the ChemStation and DataAnalysis software (Agilent, Santa Clara, CA, USA) were used.

Moreover, in order to identify other six polyphenols in BBE samples (epicatechin, catechin, syringic acid, gallic acid, protocatechuic acid, and vanillic acid) a second LC-MS method was employed [15]. For the separation of the aforementioned compounds, the same analytical column as previously specified was used. Likewise, the mobile phase consisted of a mixture of methanol: acetic acid 0.1% (v/v) and a binary gradient was used. The elution started with 3% methanol for 3 min, followed by 8% methanol until 8.5 min when 20% methanol was used and kept for the next 10 min, and then the column was rebalanced with 3% methanol. The flow rate was set at 1 mL/min and the sample injection volume was 5 µL. The MS detection mode was selected for detection of the polyphenolic compounds. The ionization on the MS system was in negative mode using an electrospray ion source (capillary +3000 V, nebulizer 60 psi (nitrogen), dry gas nitrogen at 12 L/min, dry gas temperature 360 °C). Each identified polyphenol was further quantified in BBE extracts. The results were expressed as milligrams of phenolic compound per gram of herbal material.

### 2.6. Antioxidant Activity Assays

The capacity to scavenge the DPPH, monitored according to the method described by Martins et al. [15,17] was performed by using a SPECTRO star Nano microplate reader (BMG Labtech, Offenburg, Germany). The results were expressed as Trolox equivalents (TE) per gram of dry extract (mg TE/g of dry extract).

The radical scavenging activity of the beech bark extracts against ABTS was measured according to Mocan et al. [18]. The results were expressed as milligrams of TE per gram of dry extract (mg TE/g dry extract).

In FRAP assay, the reduction of Fe^3+^-TPTZ to blue-colored Fe^2+^-TPTZ complex was monitored [19]. Briefly, the FRAP reagent was prepared by mixing ten volumes of acetate buffer (300 mM, pH 3.6), one volume of TPTZ solution (10 mM TPTZ in 40 mM HCl) and one volume of FeCl_3_ solution (20 mM FeCl_3_·6H_2_O in 40 mM HCl). The reaction mixture (25 µL sample and 175 µL FRAP reagent) was incubated for 30 min at room temperature (in the dark) and the absorbance was measured at 593 nm (SPECTROstar Nano Multi-Detection Microplate Reader with 96-well plates, BMG Labtech, Ortenberg, Germany). A Trolox^TM^ calibration curve (0.01–0.10 mg/mL) was plotted, and the results were expressed as milligrams of TE per milligram of dry extract (mg TE/mg dry extract).

### 2.7. Antidiabetic (Glucosidase inhibitory) Assay

The α-glucosidase inhibitory assay was measured according to method described previously [20,21]. In brief, 50 μL of sample diluted in 50 μL 100 mM-phosphate buffer (pH 6.8) in a 96-well microplate, were mixed with 50 μL yeast α-glucosidase for 10 min before 50 μL substrate (5 mM, p-nitrophenyl-α-D-glucopyranoside prepared in same buffer) were added. The release of p-nitrophenol was measured at 405 nm, 20 min after incubation. The blanks for test samples were prepared, and acarbose was used as a standard inhibitor, the results being expressed as IC_50_ or percentage of inhibition (where the sample was not enough active to be able to calculate an IC_50_ value for a tested concentration of 8 mg/mL) using the following formula:

Inhibition (%) = [(Abs_control_ − Abs_sample_)/Abs_control_] × 100 (1)

### 2.8. Tyrosinase Inhibitory Activity

Tyrosinase inhibitory activity of each sample was determined by method previously described by Chen et al. [22] using a SPECTROstar Nano Multi-Detection Microplate Reader with 96-well plates (BMG Labtech, Ortenberg, Germany). Samples were dissolved in water containing 5% DMSO; for each sample four wells were designated as A, B, C, D; each one contained a reaction mixture (200 µL) as follows: (A) 120 µL of 66 mM phosphate buffer solution (pH = 6.8) (PBS), 40 µL of mushroom tyrosinase in PBS (46 U/mL) (Tyr), (B) 160 µL PBS, (C) 80 µL PBS, 40 µL Tyr, 40 µL sample, and (D) 120 µL PBS, 40 µL sample. The plate was then incubated at room temperature for 10 min; after incubation, 40 µL of 2.5 mM L-DOPA in PBS solution were added in each well and the mixtures were incubated again at room temperature for 20 min. The absorbance of each well was measured at 475 nm, and the inhibition percentage of tyrosinase activity was calculated by the following equation, using a kojic acid solution (0.10 mg/mL) as a positive control:

(2) $$\%I=\frac{\left(A-B\right)-\left(C-D\right)}{\left(A-B\right)}\;\times \;100$$

### 2.9. Assay of Antimicrobial Activity

#### 2.9.1. Bacteria and Culture Conditions

For bacterial strains were used: one gram positive strain, Staphylococcus aureus (ATCC 49444), and three gram negative strains, namely *Pseudomonas aeruginosa* (ATCC 27853), *Salmonella typhimurium* (ATCC 14028) and *Escherichia coli* (ATCC 25922). The strains were cultured on Muller-Hinton agar stored at 4 °C.

#### 2.9.2. Microdilution Method

The modified microdilution technique was used to evaluate the antimicrobial activity. Cell suspensions were adjusted to a concentration of approximately 2 × 10^5^ CFU/mL in a final volume of 100 μL per well. The inoculum was stored at 4 °C for further use. Minimum inhibitory concentrations (MICs) were performed using 96-well plates. Dilutions of the extracts were carried out in wells containing 100 μL of Muller-Hinton broth and 10 μL of inoculum was added to all the wells. The microplates were incubated for 24 h at 37 °C. The MIC was detected following the addition of 20 μL (0.2 mg/mL) of resazurin solution to each well, and the plates were incubated 2 h at 37 °C. The MIC was identified as the lowest concentration that prevented the color change from blue to pink. The minimum bactericidal concentrations (MBCs) were determined by serial subcultivation of 2 μL into 96-well plates containing 100 μL of broth per well and further incubation for 48 h at 37 °C. The lowest concentration with no visible growth was defined as MBC, indicating 99.5% killing of the original inoculum.

#### 2.9.3. Antifungal Activity

Antifungal activities were investigated by using the following fungi: *Candida albicans* (ATCC 10231), *Candida parapsilosis* (ATCC 22019), and *Candida zeylanoides* (ATCC 20356). Spore suspension (1.5 × 10^5^) was obtained by washing agar plates with sterile solution (0.85% saline, 0.1% Tween 80 (v/v)), then added to each well for a final volume of 100 μL. The minimum inhibitory (MIC) and minimum fungicidal (MFC) concentrations assays were performed using the microdilution method by preparing a serial of dilutions in 96-well plates. The extracts were diluted in 0.85% saline (10 mg/mL), then added to microplates containing Broth Malt medium with inoculum and incubated for 72 h at 28 °C on a rotary shaker. The lowest concentrations without visible growth (at the binocular microscope) were defined as minimal inhibitory concentrations (MICs). The fungicidal concentrations (MFCs) were determined by serial sub-cultivation of 2 μL of tested extracts dissolved in medium and inoculated for 72 h, into microtiter plates containing 100 μL of broth per well and further incubation for 72 h at 28 °C. The lowest concentration with no visible growth was defined as MFC indicating 99.5% killing of the original inoculum. The fungicide fluconazole (Sigma F 8929, Santa Clara, CA, USA) was used as positive control (1–3500 μg/mL). All the experiments were performed in duplicate and repeated thrice. Water was used as a negative control.

### 2.10. Mutagen and Antimutagen Activity

Mutagen and antimutagenity of samples were examined using the plate incorporation method [23] described in detailed by Sarac and Sen [24]. 4-NPD (4-nitro-o-phenylenediamine) 3 µg/plate and NaN_3_ 8 µg/plate were used as positive controls for *S. thyphimurium* TA98 and *S. thyphimurium* TA100 (negative control—ethanol:water 1:1, v/v). The concentration of BBE was 5 mg/plate. The antimutagenity of the reference mutagens in the absence of the BBE was defined as 0% inhibition, and the antimutagenity was calculated according to the formula given by Ong et al., [25] as it follows: % Inhibition = [1 − T/M] × 100, where, T is the number of revertants per plate in the presence of mutagen and the BBE and M is the number of revertants per plate (without BBE) in the positive control. The data was presented as mean ± standard deviation (SD). Antimutagenity was recorded as follows: strong: 40% or more inhibition; moderate: 25%–40% inhibition; low/none: 25% or less inhibition [26].

### 2.11. Statistical Analysis

All samples were analyzed in triplicate (n = 3) and the results were expressed as the mean ± Standard Deviation (SD). The statistical analysis of the obtained experimental data was also performed by using the Modde 11.0 DoE software (Sartorius Stedim Data Analytics AB, Umeå, Sweden), which automatically calculated the statistical parameters necessary for the evaluation of the fitting quality, including the ANOVA test.

## 3. Results and Discussion

### 3.1. Fitting of the Experimental Data with the Models

The quantitative factors studied were the microwave power, with 4 levels of variation (300, 450, 600, 800 W) and the extraction time, varied over 3 levels (2, 3, 4 min). The total phenolic contents (TPCs) extracted with different solvent types (water, ethanol–water 50:50 and 80:20) were introduced in the DoE as 3 separate responses, which allowed a good comparison of their TPC extraction capacities.

As presented in Table 2, the design matrix calculated by the DoE software comprised 15 experimental runs, including three replicates that were used to confirm the experimental reproducibility, and to allow the identification of potential errors. Moreover, in order to reduce foreseeable results, the experimental runs were performed in a randomized order, dictated by the software. After performing all experimental runs, the registered response values were centralized and further introduced into the DoE matrix, where the fitting of the data has been accomplished by applying a multiple linear regression (MLR) algorithm. The fitting quality was evaluated by using the recommended, most reliable statistical parameters, i.e., the goodness of fit (R2), prediction capacities (Q2), model reproducibility given by the three performed replicates and model validity represented by the ANOVA test.

**Figure 1:**
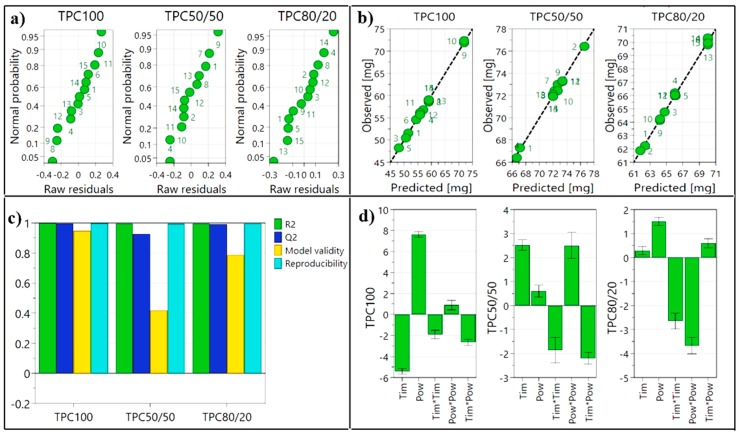
Graphical summary of (**a**) residuals normal probability; (**b**) observed vs. predicted (**c**) summary of fit; (**d**) scaled and centered coefficients (TPC100—total phenolic content in extracts obtained with water 100%, TPC50/50—total phenolic content in extracts obtained with ethanol–water 50:50, TPC80/20—total phenolic content in extracts obtained with ethanol–water 80:20).

Firstly, in order to improve the raw model by identifying and eliminating potential outliers, two graphs were generated for each response separately; the residuals plot (Figure 1a) which illustrates the residuals on a cumulative normal probability scale and the observed vs. predicted plot (Figure 1b) which allows the investigation of individual points that deviate from the diagonal line. By analyzing the two plots, outliers—marked with gray in Table 3, have been identified and eliminated, providing in this way an excellent fitted model, with high prediction capacities, as shown in the summary of fit chart (Figure 1c). In order to confirm the statistical validity of the model, the ANOVA test was performed (Supplementary file, Table S1). The registered *p* values were <0.001 for the model, describing a statistically valid design, indicating a significant impact of the factors on the responses. The *p* values registered for the lack of fit were > 0.099, showing that the model has insignificant lack of fit [27].

The generated coefficient plot (Figure 1d) allows the evaluation of factors’ (independent variables) effects over the obtained responses, this type of diagram is used to evaluate the significance and influence of the model terms, each term or interaction being represented as a scaled and centered coefficient. In the present case, the diagram, shows that the extraction time varies inversely (for water 100%) or directly (for ethanol–water 50:50) with the amount of extracted TPC. The microwave power favors TPC extraction but in different proportions.

### 3.2. Optimization of the Extraction Parameters

The fitting and analysis of the data obtained after performing all the experimental runs stipulated in the DoE matrix, led to an in depth understanding of the variables’ influence over the microwaved extraction process. Further, the optimization module of the DoE software has been used in order to calculate the optimal combination of extraction parameters, having as target the highest extraction yield obtained by using stable parameters. The optimization has been performed separately for each type of extraction solvent, the software also being able to predict not only the optimal combination of extraction parameters, but also the extracted TPC value.

After performing the stipulated optimal runs, results of the experimentally obtained TPC were compared with the DoE predicted ones, the registered recovery percent being >96% for BBE1 and close to 100% for BBE2 and BBE3, highlighting the high prediction capacity of the model. The optimal extraction parameters, DoE predicted- and experimentally-obtained TPC values are presented in Table 3.

### 3.3. Identification and Quantification of Individual Polyphenols

The optimized extracts obtained from the beech bark (water—BBE1, 50% (v/v) ethanolic—BBE2 and 80% (v/v) ethanolic—BBE3) were investigated in terms of their phytochemical profile. For the individual polyphenols identification and quantification, the previously described LC-MS/MS analytical methods were employed. The results of this analysis are summarized in Table 4 [15].

From the 18 phenolic compounds analyzed by the LC-MS/MS method, chlorogenic acid, ferulic acid and isoquercitrin, were identified in all tested BBEs. Quercetin was identified only in beech bark extract obtained with 100%water (BBE1). From the phenolic compounds identified by this method, only ferulic acid found in BBE1, was quantified (43.7 ± 5.03 µg/g dry plant material). The polyphenols (epicatechin, catechin, syringic acid, protocatechuic acid, and vanillic acid) analyzed by the other LC-MS method were identified and quantified in all tested BBEs (Table 4). Gallic acid was identified only in BBE1.

In previous studies some of the phenolic compounds obtained from beech bark were identified, including catechin, epicatechin, quercetin, taxifolin, procyanidins, syringic acid, coumaric acid [11,12].

### 3.4. Assay of the Antioxidant Activity

The antioxidant activity of optimized beech bark extracts was evaluated by DPPH, FRAP and TEAC assays. The beech bark extracts exhibited scavenging activity against all radicals as shown in Table 5. The strongest antioxidant activity is recorded for BBE2, where the strongest amount of phenols was recorded. Many bark extracts have been evaluated for their antioxidant capacities, commonly associated to their content of phenolic compounds [28,29,30]. Beech bark contains some of biologically active components, such as catechin, epicatechin, syringic acid, vanillic acid etc., indicating that the antioxidant activity of the extracts can be at least partially ascribed to these bio-components (Table 4). Grzesik et al. [31] studied the antioxidant properties of five catechins, compared with other natural or synthetic compounds. They concluded that catechins showed the strongest ABTS scavenging capacity and the strongest stoichiometry of Fe^3+^ reduction in the FRAP assay [31]. Besides the direct antioxidant properties of catechins, they may show synergistic interaction with endogenous antioxidants and act as indirect antioxidants as well [32,33].

### 3.5. Assay of the Antimicrobial Activity

The antimicrobial (antibacterial and antifungal) activity of BBEs was tested against four bacteria and three fungi, selected based on their relevance for public health. *Staphylococcus aureus* was the most sensitive strain towards all tested samples, with similar values of MIC (1.56 mg/mL) and MBC (3.12 mg/mL) (Table 6). Additionally, all tested extracts showed a good antibacterial activity on *E. coli, P. aeruginosa*, and *S. typhimurium* strains (MIC—3 mg/mL, and MBC—6 mg/mL).

The antibacterial activity of phenolic compounds has been demonstrated in various studies [4,6]. The previous study of the aqueous beech bark extract, underlined the antimicrobial activity against methicillin-resistant *S. aureus* [11].

Concerning the antifungal activity of the samples (Table 7), all tested *Candida* species, exhibited the highest sensitivity to aqueous extract of beech bark (BBE1) with 25 mg/mL MIC and 50 mg/mL MFC (Table 7). The effect of ethanolic extracts (BBE2, BBE3) on *Candida* species was absent at a concentration of 50 mg/mL.

Thus, the antibacterial activity of BBE could be attributed at least in part to phenolic compounds.

### 3.6. Antimutagenity Activity

The aim was to investigate the mutagen and antimutagen activities of BBE by the bacterial reverse mutation assay in *Salmonella typhimurium* (*S. typhimurium*) strains. The antimutagenity of BBE against *S. typhimurium* TA 98 and TA 100, was tested by comparing the numbers of induced revertants and spontaneous revertants. According with Table 8, TA98 and TA100 strains increase in the number of revertant colonies compared with the negative control when the bacteria was treated with BBE at 5 mg/plate concentration, thereby indicating an antimutagenic activity. Thus, all extracts were antimutagenic to the frameshift TA98 or TA100 *S. typhimurium* strains.

To determine the potential antimutagen activity of the BBE to prevent DNA damage by 4-NPD/NaN3 (positive mutagen/carcinogen), BBE were incubated together with 4-NPD/NaN3. The results are presented in Table 8. The percentage of inhibition of mutagen activity of 4-NPD/NaN3 (antimutagenity) of the beech bark extracts ranged from 3.6 to 17.01% in *S. typhimurium* TA98 and from 15.47 to 16.33% in *S. typhimurium* TA100. The BBE1 had the highest value for antimutagen activity (17% for *S. typhimurium* TA98 and 16.33% for *S. typhimurium* TA100). The antimutagen effect is considered low when the inhibitory effect is less than 25%, moderate when the inhibitory effect is between 25%–40% and strong when the effect is more than 45% [26,34]. Thus, based on the literature data, beech bark extracts assayed in this study may be considered to have low or no antimutagen effects.

### 3.7. In Vitro Enzyme Inhibitory Properties of Beech Bark Extracts—α-Glucosidase (Antidiabetic) and Tyrosinase Inhibitory Activity

Diabetes mellitus is a chronic, life-long disorder and is characterized by high blood glucose levels. One of the ways of treating diabetes mellitus is based on glucose absorption delay by inhibiting enzymes such as α-glucosidase in digestive organs [35]. By inhibiting α-glucosidase in the intestine, the rate of oligosaccharides hydrolysis is low, and the carbohydrate digestion process extends into the lower part of the small intestine [36]. Many studies have demonstrated the α-glucosidase inhibitory activity of some bark herbal extracts, proving their strong biochemical potential [37,38]. For example, different extracts of *Canarium tramdenum* bark were evaluated in terms of α-glucosydase inhibition showing a strong inhibitory activity. The different enriched extracts in terpenoids and phenolics appeared as a promising source of natural α-glucosidase inhibitors [38].

As shown in Table 9, the α-glucosidase inhibitory activity was determined for all BBEs and was higher in comparison with the standard acarbose (Table 9). The order of α-glucosidase inhibition is BBE3 > BBE1 > BBE2 > acarbose corresponding to IC_50_ values 38, 92, 168, and 838 µg/mL, respectively.

Tyrosinase inhibitors are compounds capable of reducing enzymatic reactions, especially from the skin, which makes them commercially relevant for cosmetic industry [39]. At a concentration of 4.025 mg/mL, inhibitory effects have been shown only for BBE2 sample. However, kojic acid (a standard inhibitor) showed an excellent tyrosinase inhibitory activity of 97.61 ± 0.24%.

## 4. Conclusions

The results of this study indicate that MAE is an efficient extraction method of phenolic compounds from beech bark. The optimum conditions which gave the highest TPC (76.57 mg GAE/g dry plant material) were reached when the microwave power was 300 W, the extraction time was 4 min, and the solvent was a mixture of ethanol–water (50:50). The practical value of TPC after a control experiment was 76.49 mg GAE/g dry plant material.

The extracts obtained in optimum conditions were characterized by HPLC-MS/MS. The identified compounds in all tested BBEs were: vanillic acid, epicatechin, catechin, protocatechuic acid, chlorogenic acid, ferulic acid, and isoquercitrin. Quercetin and gallic acid were identified only in beech bark extract obtained with 100%water. 

The beech bark extracts exhibited free-radical-scavenging activity in all assays, and were active against *S. aureus, P. aeruginosa, S. typhimurium,* and *E. coli*. All tested *Candida* species were sensitive to beech bark aqueous extract. Additionally, all extracts were active on α-glucosidase. The beech bark extracts assayed may be considered to have low or no antimutagen effects. 

This study documented that *Fagus sylvatica* L. bark possesses antioxidant activity and has α-glucosidase inhibitory effects, by several *in vitro* experiments. Ethanol and water extracts can be considered as promising sources of natural antioxidants, antimicrobian agents, and α-glucosidase inhibitors. The isolation of bioactive constituents and investigations on several biomedical properties of *F. sylvatica* bark should be conducted in future experiments.

## Figures and Tables

**Table 1:** Variables, symbols, and levels of variation used in the experimental design.

Variables	Symbol	Level of Variation			
**Independent Variables (Factors)**					
Extraction time (min)	X1	2		3	4
Microwave power (W)	X2	300	450	600	800
**Dependent Variables (Responses)**					
Extraction solvent-water	BBE 1				
Extraction solvent—50:50 ethanol–water	BBE 2				
Extraction solvent—80:20 ethanol–water	BBE 3				

Notes: mg GAE/g = gallic acid equivalents per gram plant material.

**Table 2:** Matrix of experimental design and experimental results for total phenolic content (TPC).

Sample Code	Run Order	Independent Variables (Factors)		Dependent Variables (Responses)		
		Extraction Time (min)	Microwave Power (W)	BBE 1	BBE 2	BBE 3
				(TPC mg GAE/g Dry Plant Material ± SD)		
N1	1	2	300	51.53	67.27	62.26
N2	6	4	300	**47.44**	76.46	61.87
N3	3	3	300	50.53	**69.59**	64.77
N4	2	2	450	56.79	66.43	66.20
N5	7	4	450	48.19	**77.53**	66.00
N6	5	3	450	54.55	**70.32**	**67.86**
N7	11	4	600	**52.79**	72.43	*70.95*
N8	10	3	600	58.61	72.06	70.20
N9	8	2	800	71.80	73.02	64.12
N10	14	2	800	72.31	72.46	64.27
N11	4	4	800	56.15	73.23	65.97
N12	12	4	800	55.68	73.32	66.07
N13	9	3	600	58.91	72.09	69.81
N14	15	3	600	59.00	71.91	70.33
N15	13	3	600	59.10	71.92	69.93

Notes: values with bold—outliers, TPC—total phenolic content expressed as mg GAE/g—gallic acid equivalents per dry plant material. BBE 1—extracts obtained with water 100%, BBE 2 extracts obtained with ethanol–water 50:50, BBE 3 extracts obtained with ethanol–water 80:20. Each value is the mean ± SD of three independent measurements.

**Table 3:** Optimal extraction parameters, DoE predicted and experimental obtained values of the extracted TPC.

Extraction Solvent	Symbol	Extraction Time (min)	Microwave Power (W)	DoE Predicted Values	Experimental Obtained Values	Recovery (%)
				(TPC mg GAE/g Plant Material)		
water	BBE 1	2	800	72.05	69.76	96.82
50:50 ethanol–water	BBE 2	4	300	76.55	76.31	99.69
80:20 ethanol–water	BBE 3	3.1	600	70.09	69.75	99.51

Notes: BBE 1—extracts obtained with water 100%, BBE 2—extracts obtained with ethanol–water 50:50, BBE 3—extracts obtained with ethanol–water 80:20.

**Table 4:** Quantitative (µg/g dry plant material) evaluation of the recovery of main bioactive compounds in samples of BBE.

Sample Code	Bioactive Compounds					
	(−)-Epicatechin	(+)-Catechin	Syringic Acid	Gallic Acid	Protocatechuic Acid	Vanillic Acid
BBE1	22.7 ± 2.72	300.7 ± 44.22	24.2 ± 3.02	1.9 ± 0.14	3.3 ± 0.29	49.9 ± 7.28
BBE2	39.6 ± 3.24	577.4 ± 56.58	7.5 ± 0.78	NF	6.2 ± 0.41	18.0 ± 1.52
BBE3	33.4 ± 3.55	465.1 ± 51.62	5.8 ± 0.68	NF	5.7 ± 0.74	16.1 ± 1.55

Notes: NF—not found, below limit of detection; BBE1—extracts obtained with water 100%, BBE2—extracts obtained with ethanol–water 50:50, BBE3—extracts obtained with ethanol–water 80:20. Data are shown as mean ± standard deviation (SD).

**Table 5:** Antioxidant activity of optimized beech bark extracts.

Sample Code	TPC mg GAE/g of Dry Extract	DPPH mg TE/g of Dry Extract	TEAC mg TE/g of Dry Extract	FRAP mg TE/g of Dry Extract
**BBE 1**	69.76 ± 1.54	676.29 ± 19.80	472.08 ± 67.07	625.13 ± 9.62
**BBE 2**	76.49 ± 2.41	741.43 ± 59.44	619.85 ± 20.75	783.24 ± 31.24
**BBE 3**	69.86 ± 1.04	505.02 ± 42.02	464.41 ± 37.42	592.84 ± 44.02

TPC—total phenolic content, DPPH—2,2-diphenyl-1-picrylhydrazyl, TEAC—Trolox equivalent antioxidant capacity, FRAP—ferric reducing ability of plasma, BBE1- extracts obtained with water 100%, BBE2—extracts obtained with ethanol–water 50:50, BBE3—extracts obtained with ethanol–water 80:20 ± Standard deviation.

**Table 6:** Antibacterial activity (mg/mL) of the beech bark extracts (BBE).

Sample Code	Bacteria			
	*Staphylococcus aureus* (ATCC 49444)	*Escherichia coli* (ATCC 25922)	*Pseudomonas aeruginosa* (ATCC 27853)	*Salmonella typhimurium* (ATCC 14028)
Minimum Inhibitory Concentration (MIC)				
BBE1	1.56	3	3	3
BBE2	1.56	3	3	3
BBE3	1.56	3	3	3
Minimum Bactericidal Concentration (MBC)				
BBE1	3.12	6	6	6
BBE2	3.12	6	6	6
BBE3	3.12	6	6	6

Notes: BBE1—extracts obtained with water 100%, BBE2—extracts obtained with ethanol–water 50:50, BBE3—extracts obtained with ethanol–water 80:20.

**Table 7:** Antifungal activity (mg/mL) of the beech bark extracts (BBE).

Sample Code	Fungi		
	*Candida albicans* (ATCC 10231)	*Candida parapsilosis* (ATCC 22019)	*Candida zeylanoides* (ATCC 20356)
Minimum Inhibitory Concentration (MIC)			
BBE1	25	25	25
BBE2	NF	>50	>50
BBE3	NF	>50	>50
Minimum Fungicidal Concentration (MFC)			
BBE1	50	50	50
BBE2	NF	>50	>50
BBE3	NF	>50	>50

Notes: BBE1—extracts obtained with water 100%, BBE2—extracts obtained with ethanol–water 50:50, BBE3—extracts obtained with ethanol–water 80:20.

**Table 8:** Antimutagen properties of beech bark extracts (5 mg/plate) on *S. typhimurium* TA98 and TA100 bacterial strains.

Test Item	Number of Revertants			
	TA 98		TA100	
	Mean ± SD	Inhibition %	Mean ± SD	Inhibition %
Negative Control	9.25 ± 3.6 ^a^		9.25 ± 2.4 ^b^	
BBE1 ^a^	161 ± 3.6	17,01	292 ± 6.4	16,33
BBE2 ^a^	187 ± 4.4	3.60	295 ± 6.4	15.47
BBE3 ^a^	172 ± 3.8	11.34	294 ± 6.2	15,75
4-NPD ^c^	193 ± 3.4	-	-	-
NaN_3_^c^	-	-	349 ± 15.22	-

Notes: ^a^ BBE1—extracts obtained with water 100%, BBE2—extracts obtained with ethanol–water 50:50, BBE3—extracts obtained with ethanol–water 80:20; ^b^ Values expressed are means ± SD of three replications; ^c^ 4-NPD and NaN_3_ were used as positive controls for *S. thyphimurium* TA98 and TA100 strains, respectively.

**Table 9:** Enzyme inhibitory effects of beech bark extracts.

Nr. crt.	Sample	Glucosidase Inhibition (IC_50_ µg/mL)	Tyrosinase Inhibition (PI—4.025 mg/mL)
1.	BBE1	92	NF
2.	BBE2	168	45.99 ± 5.26%
3.	BBE3	38	NF
4.	Acarbose	838	-
5	Kojic acid (1 mg/mL)	-	97.61 ± 0.24%

Notes: BBE1—extracts obtained with water 100%, BBE2—extracts obtained with ethanol–water 50:50, BBE3—extracts obtained with ethanol–water 80:20, PI—procent of inhibition, NF—not found.

## Supplementary Materials

The following are available online at https://www.mdpi.com/2076-3921/8/9/417/s1, Table S1: ANOVA—complete summary of the regression and residual analysis.
