# Supplementary material for: Biological and Chemical Insights of Beech (Fagus sylvatica L.) Bark: A Source of Bioactive Compounds with Functional Properties
Source: Antioxidants (Basel). 2019 Sep 19;8(9):417. doi: 10.3390/antiox8090417 (PMC6769934; doi:10.3390/antiox8090417)
Supplement: Supplementary file 1 [file antioxidants-08-00417-s001.pdf]

Supplementary Material

Table S1 - ANOVA – complete summary of the regression and residual analysis

	DF	SS	MS (variance)	F	p	SD
**BBE1**						
Total	13	44,248.1	3403.7			
Constant	1	43,633.4	43633.4			
Total corrected	12	614.64	51.22			7.15682
Regression	5	614.233	122.847	2112.44	**0.000**	11.0836
Residual	7	0.407077	0.0581539			0.241151
Lack of Fit	2	0.032481	0.0162404	0.216771	**0.812**	0.127438
**BBE2**						
	**DF**	**SS**	**MS (variance)**	**F**	**p**	**SD**
Total	12	62,084.4	5173.7			
Constant	1	62,006.6	62006.6			
Total corrected	11	77.8445	7.07677			2.66022
Regression	5	77.5041	15.5008	273.225	**0.000**	3.93711
Residual	6	0.340397	0.0567328			0.238186
Lack of Fit	1	0.153448	0.153448	4.10399	**0.099**	0.391724
**BBE3**						
	**DF**	**SS**	**MS (variance)**	**F**	**p**	**SD**
Total	13	57,235.1	4402.7			
Constant	1	57,130.7	57130.7			
Total corrected	12	104.37	8.69747			2.94915
Regression	5	104.105	20.821	551.094	**0.000**	4.56301
Residual	7	0.264469	0.0377813			0.194374
Lack of Fit	2	0.076544	0.0382718	1.01827	**0.426**	0.195632

Abbreviations: DF – degrees of freedom, SS – sum of squares, MS – mean square (=SS/DF), F-distribution value, p – probability, SD – standard deviation.
